# Unveiling the Hidden: Successful Surgical Management of a Rare Case of Chronic Perineal Sinus Tract

**DOI:** 10.7759/cureus.83718

**Published:** 2025-05-08

**Authors:** Ajay Nayagam P, Suganya P, Samir Ahmad

**Affiliations:** 1 Department of General Surgery, Sree Balaji Medical College and Hospital, Chennai, IND

**Keywords:** chronic, fibrotic changes, labial sinus, magnetic resonance imaging fistulogram, sinus tract, surgical excision

## Abstract

Sinus tracts are rare in the perineal region, and they present as chronic non-healing sinus tracts. This is a case report that aims to showcase the surgical management of a chronic sinus tract. A 26-year-old female presented with a history of a non-healing sinus tract below the right vulva for the last seven years. There was no significant improvement despite medical management. A curvilinear sinus tract with fibrotic changes in the right perineal region with no communication with the anal canal or vagina was seen in the MRI. Embarrassment and hesitation to seek early surgical help result in infection, formation of abscess, scarring, and perineal disfigurement. Understanding the disease's etiology, clinical presentation, and treatment options for this condition is important for effective treatment. A successful outcome was obtained when surgical excision of the sinus tract with primary closure was done. This case discusses the importance of surgical intervention in a chronic perineal sinus tract refractory to medical management.

## Introduction

Sinus tracts are abnormal, narrow, epithelium-lined channels that connect a deep-seated focus of infection or inflammation to the skin surface. They typically arise due to chronic inflammatory processes, persistent infections, or immune-mediated tissue breakdown. Common causes include conditions such as hidradenitis suppurativa, a chronic skin disorder characterized by recurrent, painful nodules and abscesses in apocrine gland-bearing areas, and pilonidal disease, in which hair and debris become embedded in the skin, often near the natal cleft, leading to infection and tract formation [1]. Autoimmune disorders like systemic lupus erythematosus (SLE) may also contribute to sinus tract development through chronic immune-mediated tissue damage, vasculitis, and impaired wound healing, which promote persistent ulceration and sinus formation. Early recognition and thorough evaluation of sinus tracts are critical to identifying the underlying etiology and guiding appropriate management.

Although sinus tracts can arise in various anatomical locations, their occurrence in the perineal region is particularly uncommon. Perineal sinus tracts are often persistent, painful, and resistant to conservative management, significantly affecting the patient's quality of life. While some cases respond to antibiotics and local wound care, chronic and recurrent lesions typically necessitate surgical excision. Advanced imaging modalities, such as MRI or ultrasonography, are instrumental in delineating the extent of the tract and guiding appropriate treatment.

This case report highlights the diagnostic and therapeutic challenges in managing a rare chronic perineal sinus tract, compounded by delayed healthcare-seeking behavior. It underscores the importance of a multidisciplinary approach involving medical management, imaging, and timely surgical intervention. Furthermore, this case adds to the limited literature on rare presentations of sinus tracts and emphasizes the need for heightened clinical suspicion and individualized treatment strategies to achieve optimal outcomes [2,3].

## Case presentation

A 26-year-old female patient presented with a seven-year history of a persistent sinus tract located in the right perineal region. Despite multiple attempts at conservative medical management, including antibiotics and local wound care, the lesion remained non-healing. Microbiological cultures obtained prior to antibiotic therapy revealed the presence of *Staphylococcus aureus*, guiding the choice of targeted antibiotics. However, no complete resolution was achieved. The patient denied any history of trauma, infection, prior surgical interventions, or systemic illnesses. No associated comorbidities or congenital abnormalities were noted. Chronic friction and occlusion in the perineal region were considered possible contributing factors.

**Figure 1:**
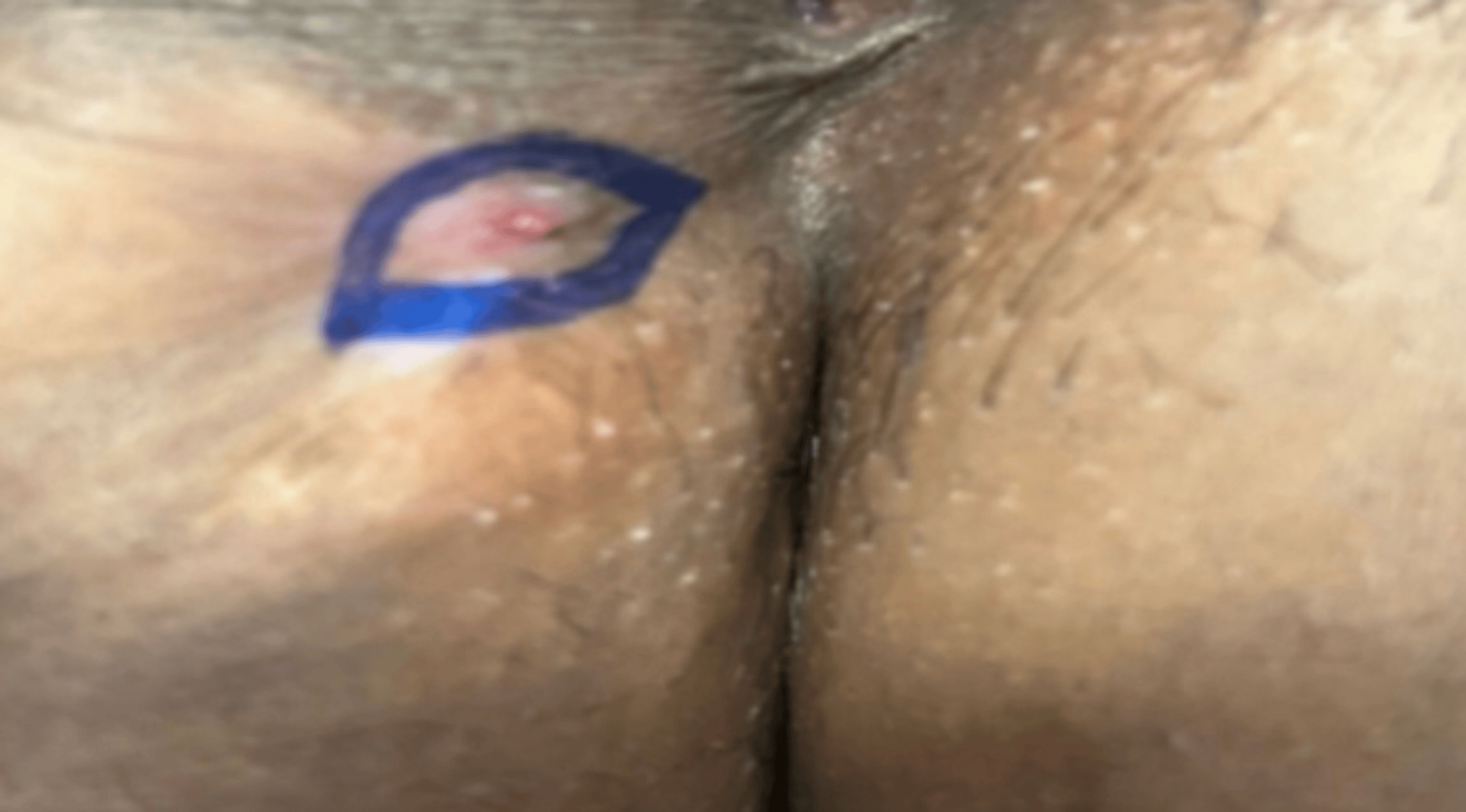
Gross image showing cutaneous opening with surrounding fibrosis in the subcutaneous plane of the right side of the perineal region (blue ellipse).

On clinical examination, a cutaneous opening with surrounding fibrosis was noted in the subcutaneous plane of the perineal region (right side) as shown in Figure 1. The lesion was tender on palpation, and although no active purulent discharge was present, signs of chronic inflammation were evident.

**Figure 2:**
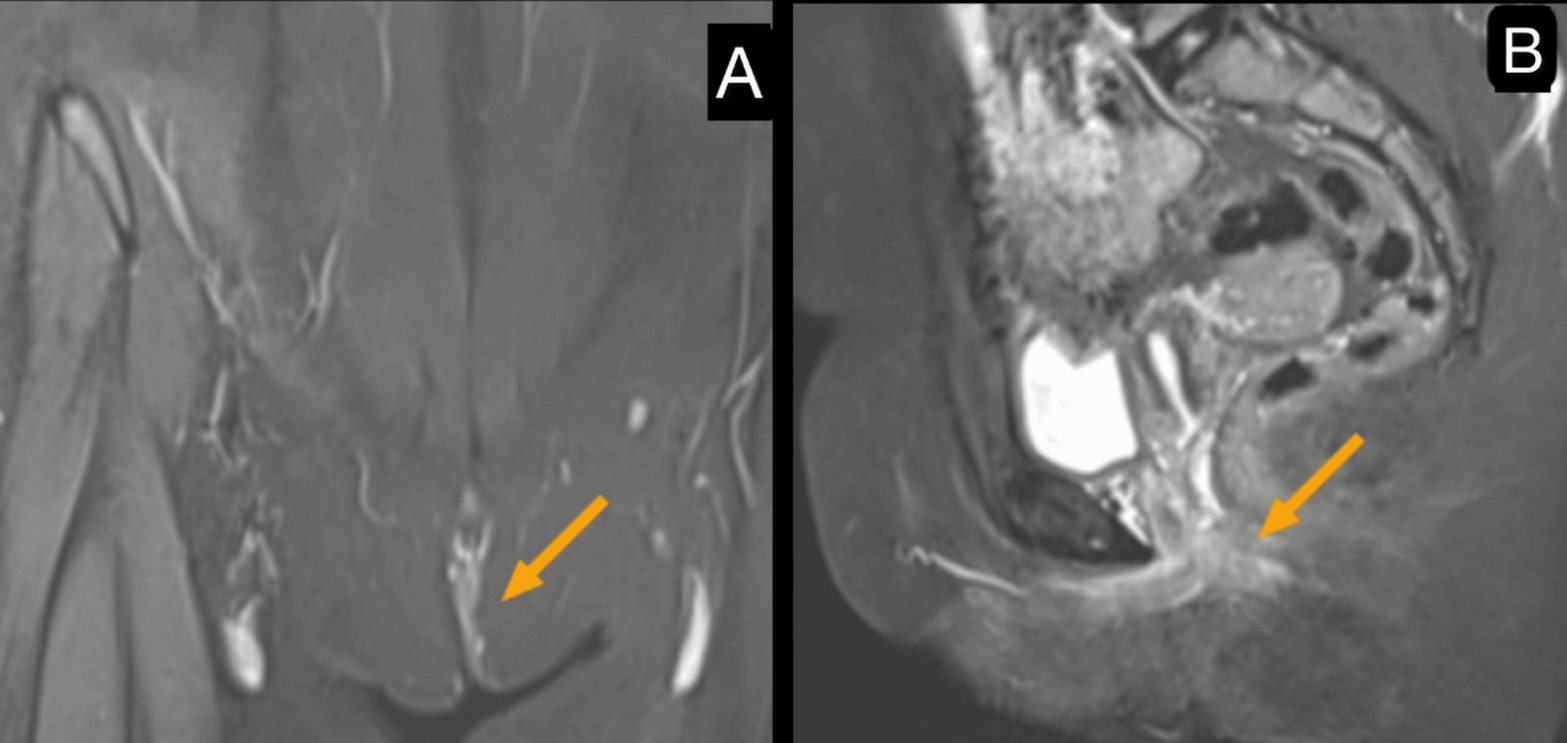
MRI fistulogram shows a curvilinear sinus tract with surrounding fibrotic changes in the subcutaneous plane of the right perineum (yellow arrow) (A) and no evidence of an abscess, mass formation, or communication with adjacent structures (yellow arrow) (B).

An MRI of the perineum revealed a curvilinear sinus tract with surrounding fibrotic changes in the subcutaneous plane of the right perineal region (Figure 2A). Importantly, there was no evidence of an abscess, mass formation, or communication with adjacent structures, ruling out potential underlying conditions such as a fistula or connection to the genitourinary system (Figure 2B).

**Figure 3:**
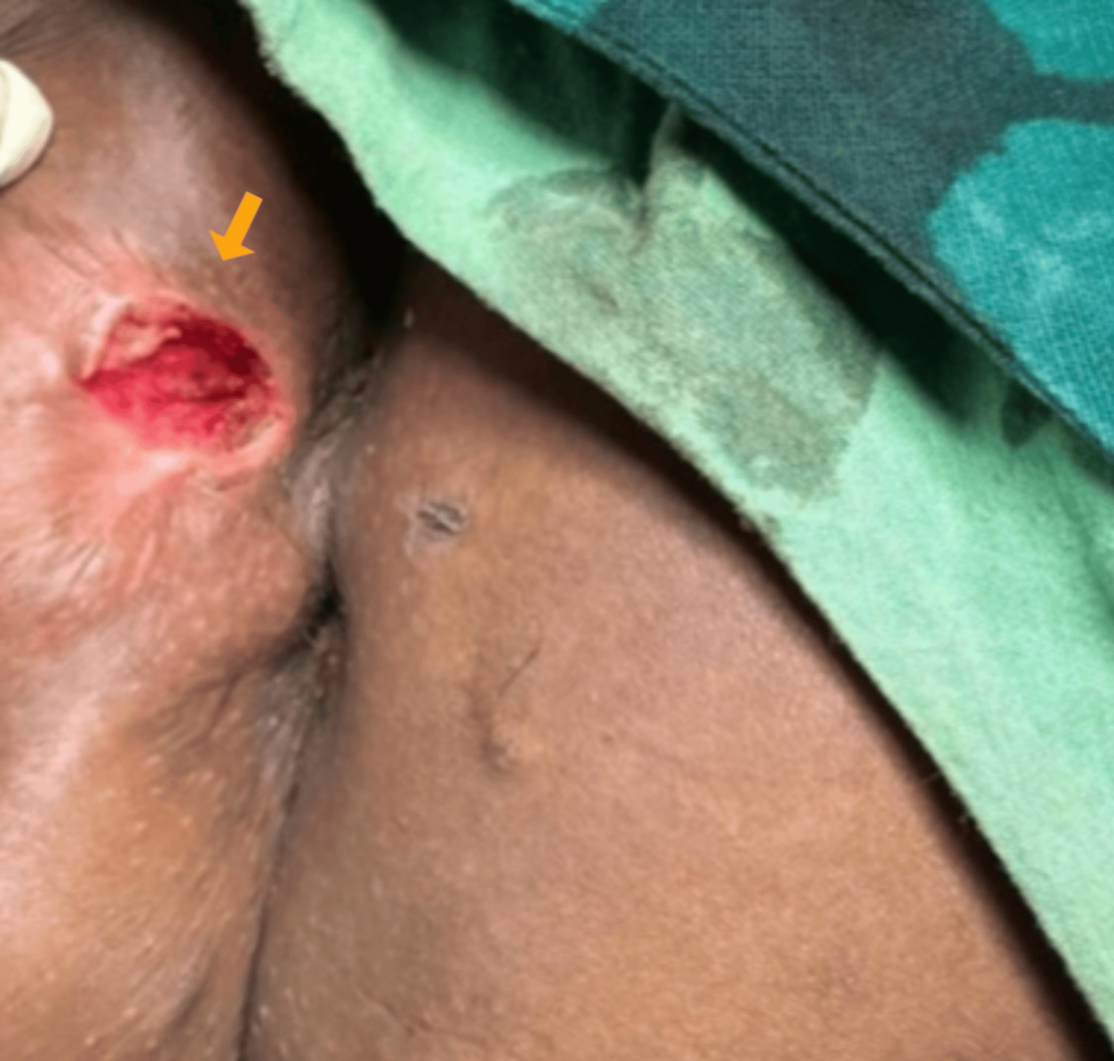
The postoperative image shows complete excision of the sinus tract, ensuring the removal of all fibrotic and diseased tissue (yellow arrow).

Due to the chronic nature of the lesion and its lack of response to medical treatment, surgical excision was planned. Under general anesthesia, complete excision of the sinus tract was performed, ensuring the removal of all fibrotic and diseased tissue (Figure 3). Primary closure of the wound was achieved following meticulous debridement. The postoperative course was uneventful, with the patient managed effectively with analgesics, antibiotics, and standard wound care.

**Figure 4:**
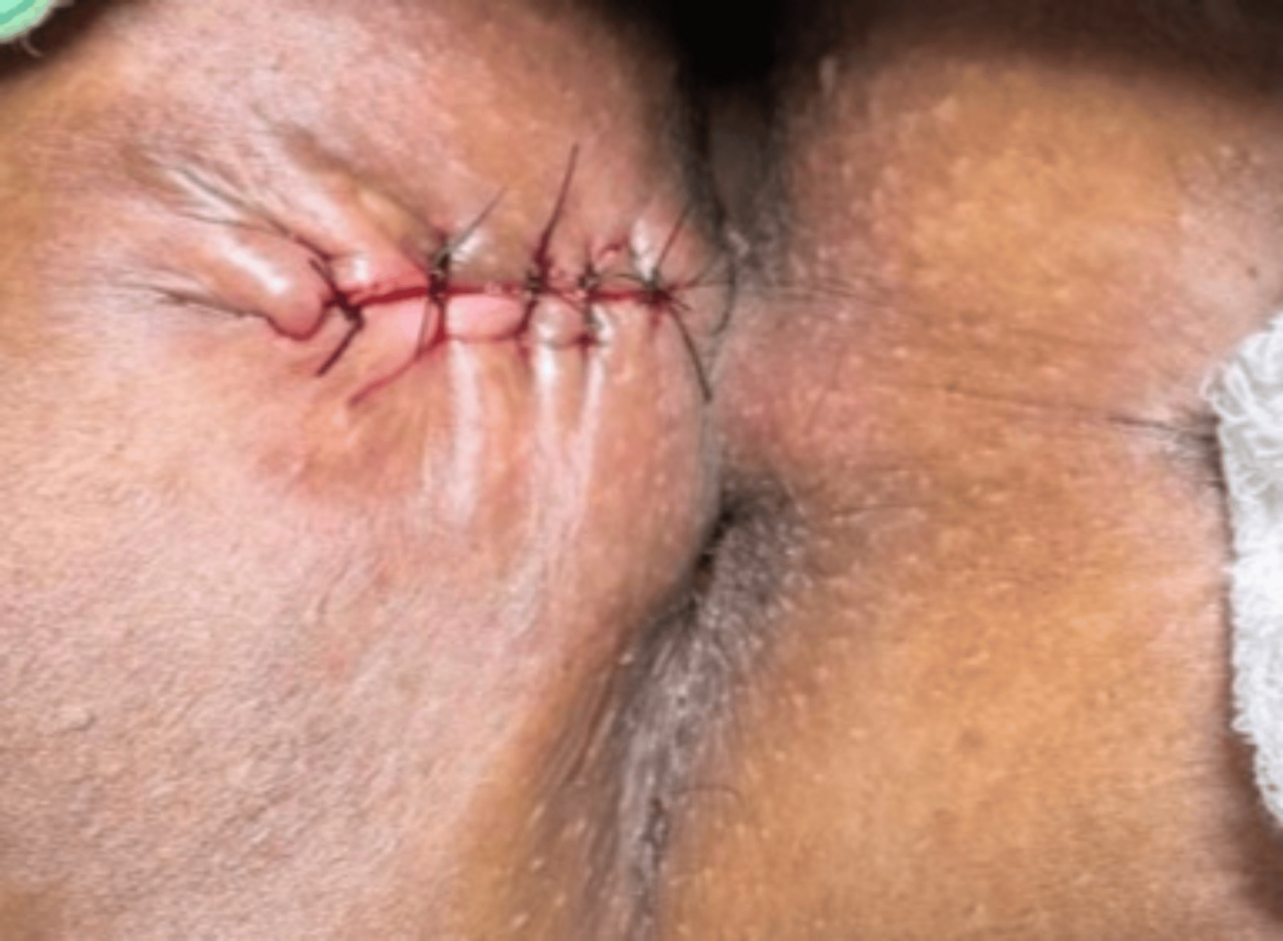
The gross image at follow-up shows the wound had healed satisfactorily with no evidence of recurrence or complications.

At the four-week follow-up, the wound had healed satisfactorily with no evidence of recurrence or complications (Figure 4). Although the follow-up duration was limited, early postoperative recovery was uneventful, and no signs of recurrence were clinically evident during this period. This case highlights the significance of detailed preoperative imaging in surgical planning, particularly in perineal sinus tracts where anatomical considerations are critical. The successful outcome of this case underscores that, in select patients with chronic perineal sinus tracts lacking deeper communication, primary surgical excision with closure is a definitive and effective treatment strategy, offering excellent clinical outcomes with minimal morbidity.

## Discussion

Chronic perineal sinus tracts are rare and often pose significant challenges in management, particularly when resistant to medical therapy. A sinus tract is defined as an abnormal channel that connects an infected area or abscess to an epithelial surface, often associated with chronic inflammation. Unlike fistulas, which typically connect two epithelial-lined surfaces, sinus tracts usually have a blind-ended opening [3,4]. Their occurrence in the perineal region is uncommon, necessitating thorough diagnostic evaluation and individualized treatment approaches.

To assess the extent of the sinus tract and rule out communication with adjacent structures such as the anal canal or vagina, imaging plays a crucial role. MRI is considered the gold standard for evaluating soft tissue involvement and confirming the absence of fistulous connections. In this case, MRI findings demonstrated a localized sinus tract without deeper communication, allowing for direct surgical intervention [5,6].

Conservative management, including antibiotics, local wound care, and avoidance of potential irritants, is typically the first line of treatment in the acute stage. However, when medical therapy fails, surgical excision remains the definitive management strategy. The key surgical objective is the complete removal of the sinus tract along with surrounding fibrotic tissue to prevent recurrence [7]. Histopathological evaluation often reveals non-specific chronic inflammation, as seen in this case.

Postoperative care is essential to ensure optimal healing and minimize complications. Patients are advised to avoid strenuous physical activities, heavy lifting, and sexual intercourse for a specified period. Additionally, the use of tampons and intravaginal devices is restricted to prevent undue pressure on the healing tissue [8,9].

Sinus tracts can develop in various anatomical regions, each with unique management considerations [10]. Table 1 compares labial sinus tracts with more common forms, such as pilonidal and perianal sinus tracts.

**Table 1:** Comparison of various sinus tracts Table compiled by the authors based on synthesis of clinical experience and published literature; Sources: [3–8].

Etiology	Chronic inflammation, congenital factors	Pilonidal sinus infection	Anal gland infection
Common symptoms	Pain, fibrosis, and discharge	Pain, swelling, recurrent abscesses	Perianal pain, pus drainage
Diagnostic imaging	MRI	Ultrasound, MRI	MRI, endoanal ultrasound
Treatment	Excision with primary closure	Excision with secondary healing or flap closure	Fistulotomy, drainage
Recurrence rate	Low with complete excision	Moderate to high	Variable depending on complexity

A review of recent case reports on perineal sinus tracts reveals that surgical excision is the preferred treatment approach. Previous reports have demonstrated high success rates with complete excision and primary closure, similar to our findings. Table 2 summarizes findings from similar cases. The existing literature on chronic perineal sinus tracts is limited primarily to isolated case reports. As summarized in Table 2, Batool et al. (2023) [1], Manoj et al. (2008) [2], and Anderson and Turnbull (1967) [11] each reported successful outcomes with no recurrence following excision and primary closure, with or without preoperative imaging. Although these are single-patient studies, the uniformity in outcome, particularly in cases where MRI was utilized to confirm the absence of deeper extensions, supports our conclusion that complete excision with primary closure can be an effective and definitive treatment in appropriately selected patients. This highlights a clear need for larger cohort studies to validate these findings more robustly.

**Table 2:** Review of literature of recent case reports on perineal sinus tract management

Study	Number of cases	Imaging used	Treatment approach	Recurrence
Batool et al. (2023) [1]	1	MRI	Excision and primary closure	0%
Manoj et al. (2008) [2]	1	Not reported	Excision and primary closure	0%
Anderson and Turnbull et al. (1976) [11]	1	MRI	Excision and primary closure	0%

Sinus tracts are often graded based on their complexity and associated anatomical involvement. While no specific classification exists for perineal sinus tracts, perianal sinus tracts are categorized into simple and complex types based on their communication with deeper structures. Applying similar principles to labial sinus tracts can help guide management [12, 13].

Future studies should focus on more comprehensive data collection, including detailed imaging protocols, patient demographics, and long-term outcomes. A standardized follow-up period is essential to assess recurrence rates more accurately and inform optimal management strategies for chronic perineal sinus tracts.

## Conclusions

This case underscores the significance of preoperative imaging in evaluating chronic perineal sinus tracts to rule out deeper anatomical communication before proceeding with surgical management. In this patient, MRI was instrumental in confirming the absence of an underlying fistula or genitourinary involvement, allowing for definitive surgical excision with primary closure. Complete removal of the sinus tract and surrounding fibrotic tissue ensured optimal healing, with no recurrence or complications during follow-up. The successful outcome in this case supports the effectiveness of primary excision with closure in non-complex perineal sinus tracts. However, based on the limitations of this study, including short follow-up and a small sample size, further studies with longer follow-up and larger cohorts are necessary to confirm the recurrence rates and identify key factors contributing to successful outcomes. Adherence to reporting guidelines and the inclusion of comprehensive data are critical to strengthening the evidence base for this treatment strategy. This case further emphasizes the importance of individualized surgical planning and contributes to the growing literature on rare presentations of sinus tracts in the perineal region.
